# Extracorporeal Shockwave Therapy for Mid-portion and Insertional Achilles Tendinopathy: A Systematic Review of Randomized Controlled Trials

**DOI:** 10.1186/s40798-022-00456-5

**Published:** 2022-05-13

**Authors:** Marc A. Paantjens, Pieter H. Helmhout, Frank J. G. Backx, Faridi S. van Etten-Jamaludin, Eric W. P. Bakker

**Affiliations:** 1 Sports Medicine Centre, Training Medicine and Training Physiology, Royal Netherlands Army, Utrecht, The Netherlands; 2 Centre of Excellence, Training Medicine and Training Physiology, Royal Netherlands Army, Utrecht, The Netherlands; 3 Department of Rehabilitation, Physical Therapy Science and Sports, University Medical Center Utrecht, Utrecht, The Netherlands; 4 Research Support, Medical Library, Amsterdam UMC, University of Amsterdam, Amsterdam, The Netherlands; 5 Division EPM, Department Epidemiology and Data Science, University Medical Center Amsterdam, Amsterdam, The Netherlands

**Keywords:** Achilles tendinopathy, Mid-portion Achilles tendinopathy, Insertional Achilles tendinopathy, Extra corporeal shockwave therapy, Sports medicine

## Abstract

**Background:**

Extracorporeal shockwave therapy (ESWT) is used commonly to treat pain and function in Achilles tendinopathy (AT). The aim of this study was to synthesize the evidence from (non-) randomized controlled trials, to determine the clinical effectiveness of ESWT for mid-portion Achilles tendinopathy (mid-AT) and insertional Achilles tendinopathy (ins-AT) separately.

**Methods:**

We searched PubMed/Medline, Embase (Ovid), and Cochrane Central, up to January 2021. Unpublished studies and gray literature were searched in trial registers (ACTRN, ChiCTR, ChiCtr, CTRI, DRKS, EUCTR, IRCT, ISRCTN, JPRN UMIN, ClinicalTrials.gov, NTR, TCTR) and databases (OpenGrey.eu, NARCIS.nl, DART-Europe.org, OATD.org). Randomized controlled trials (RCTs) and non-randomized controlled clinical trials (CCTs) were eligible when investigating the clinical effectiveness of ESWT for chronic mid-AT or chronic ins-AT. We excluded studies that focused on treating individuals with systemic conditions, and studies investigating mixed cohorts of mid-AT and ins-AT, when it was not possible to perform a subgroup analysis for both clinical entities separately. Two reviewers independently performed the study selection, quality assessment, data extraction, and grading of the evidence levels. Discrepancies were resolved through discussion or by consulting a third reviewer when necessary.

**Results:**

We included three RCTs on mid-AT and four RCTs on ins-AT. For mid-AT, moderate quality of evidence was found for the overall effectiveness of ESWT compared to standard care, with a pooled mean difference (MD) on the VISA-A of 9.08 points (95% CI 6.35–11.81). Subgroup analysis on the effects of ESWT additional to standard care for mid-AT resulted in a pooled MD on the VISA-A of 10.28 points (95% CI 7.43–13.12). For ins-AT, we found very low quality of evidence, indicating that, overall, ESWT has no additional value over standard care, with a standardized mean difference (SMD) of − 0.02 (95% CI − 0.27 to 0.23). Subgroup analysis to determine the effect of ESWT additional to standard care for ins-AT showed a negative effect (SMD − 0.29; 95% CI − 0.56 to − 0.01) compared to standard care alone.

**Conclusions:**

There is moderate evidence supporting the effectiveness of ESWT additional to a tendon loading program in mid-AT. Evidence supporting the effectiveness of ESWT for ins-AT is lacking.

*Trial Registration*: PROSPERO Database; No. CRD42021236107.

**Supplementary Information:**

The online version contains supplementary material available at 10.1186/s40798-022-00456-5.

## Key Points

- Adding extracorporeal shockwave therapy to a tendon loading program for mid-portion Achilles tendinopathy results in a clinically important improvement on the VISA-A questionnaire.
- Extracorporeal shockwave therapy seems to be ineffective for the treatment of insertional Achilles tendinopathy.

## Background

Chronic Achilles tendinopathy (AT) is a clinical condition characterized by pain, swelling, and decreased performance [1]. AT can be divided into mid-portion Achilles tendinopathy (mid-AT) and insertional Achilles tendinopathy (ins-AT). Mid-AT is more common (55–65%) than ins-AT (20–25%) [2]. AT occurs most frequently between the ages of 40–59 years [3] and is particularly prevalent in athletes, especially in runners [4].

Mechanical loading regimes are currently the standard of care for subjects with AT [4, 5]. Eccentric exercises have been considered a superior intervention, but recent studies conclude that various loading programs seem equally effective, regardless of contraction type [5–7]. Following inception of a loading program, pain and function may already improve after 2 weeks with results peaking at 12 weeks [8]. At 5-year follow-up, however, a significant portion of patients has not responded adequately to a loading strategy [9, 10], and up to half of all patients seek alternative treatment [9].

Extracorporeal shockwave therapy (ESWT) is used as a secondary conservative treatment for refractory tendinopathies [11–13]. It is thought that ESWT can influence the pathophysiological processes in various musculoskeletal conditions [14], and, by this, decrease pain and improve function in AT [4, 15]. ESWT can be used as a monotherapy [16], but is usually part of a multimodal treatment strategy [11], and is considered to improve long-term outcomes when combined with eccentric exercises [17]. ESWT is reported to be safe [18, 19] and (cost)effective for patients with persistent AT who have low responsiveness to standard care [11, 19], but the evidence is conflicting [11, 12, 20, 21].

To our current knowledge, no systematic reviews so far have included only experimental studies to review the effectiveness of ESWT for mid-AT and ins-AT separately. Therefore, we aimed to synthesize the evidence from (randomized) controlled studies to determine the clinical effectiveness of ESWT, either as a monotherapy or as an additional intervention for both chronic mid-AT and ins-AT.

## Methods

### Protocol and Registration

This systematic review was conducted according to the Preferred Reporting Items for Systematic Reviews and Meta-Analyses (PRISMA) guidelines [22] and the Cochrane Handbook for Systematic Reviews of Interventions [23]. To enhance validity and reduce unintentional duplication of effort, the study protocol was registrated in the International Prospective Register of Systematic Reviews (PROSPERO) under registration number: CRD42021236107 (https://www.crd.york.ac.uk/prospero/).

### Eligibility Criteria

#### Types of Studies

Designs eligible for inclusion were: (1) randomized controlled clinical trials (RCTs) and (2) non-randomized controlled clinical trials (CCTs).

#### Types of Participants

Studies were eligible if ESWT was used to treat patients of 18 years and older, with a clinical or radiological confirmed diagnosis of either mid-AT or ins-AT, and whose symptoms were present for at least three months. We excluded studies that focused on treating individuals with systemic conditions (e.g., rheumatoid arthritis and diabetes mellitus). Studies investigating the clinical effectiveness of ESWT in mixed cohorts of mid-AT and ins-AT were also excluded when results were not presented separately for both conditions and were also not available after contacting the authors, preventing subgroup analysis for mid-AT and ins-AT separately.

#### Types of Interventions

Two types of ESWT are common in musculoskeletal practice: focused extracorporeal shockwave therapy (F-ESWT) and radial extracorporeal shockwave therapy (R-ESWT). Both treatments are commonly applied for treating tendinopathies [11, 19]. We included studies that either used F-ESWT or R-ESWT, as a monotherapy or as an additional intervention, regardless of energy level or numbers of shockwave treatments administered.

#### Types of Comparisons

Studies investigating the efficacy of shockwave compared to different surgical and conservative interventions were eligible (e.g., tendon loading programs, surgical techniques, injections or dry needling, oral medication, placebo interventions, different shockwave modalities, or other commonly used non-surgical interventions for AT).

#### Types of Outcome Measures

Studies that used validated and reliable outcome measures to assess the clinical effectiveness of ESWT in multiple domains representing functional improvement, pain reduction, and self-perceived recovery were eligible for inclusion, such as the Victorian Institute of Sports Assessment—Achilles (VISA-A) questionnaire, the numeric rating scale for pain (NRS) or visual analogue scale for pain (VAS), and the global perceived effect.

All steps in this review were independently performed by two reviewers (MP and PH). Differences were resolved by discussion. When disagreement persisted, the opinion of a third reviewer (EWP) was decisive.

### Search Strategy

#### Electronic Databases and Reference Lists

With the assistance of a medical librarian of the Amsterdam University Medical Center (UMC), we developed an extensive search strategy. The following databases were searched from inception up to 21st January 2021: Medline, Embase, and Cochrane. The search strategy is reported in Additional file 1: Appendix I.

#### Hand Searching

Reference lists of the included articles were manually checked for additional eligible studies. If the information provided by full-text articles led to uncertainty regarding possible inclusion, the original authors were contacted for clarification.

#### Unpublished Data and Gray Literature

We also searched for unpublished studies and gray literature [24] in trial registers (ACTRN, ChiCTR, ChiCtr, CTRI, DRKS, EUCTR, IRCT, ISRCTN, JPRN UMIN, ClinicalTrials.gov, NTR, TCTR), and databases (OpenGrey.eu, NARCIS.nl, DART-Europe.org, OATD.org). No language restrictions were applied. Both published and unpublished studies were eligible.

### Study Selection

**Fig. 1:**
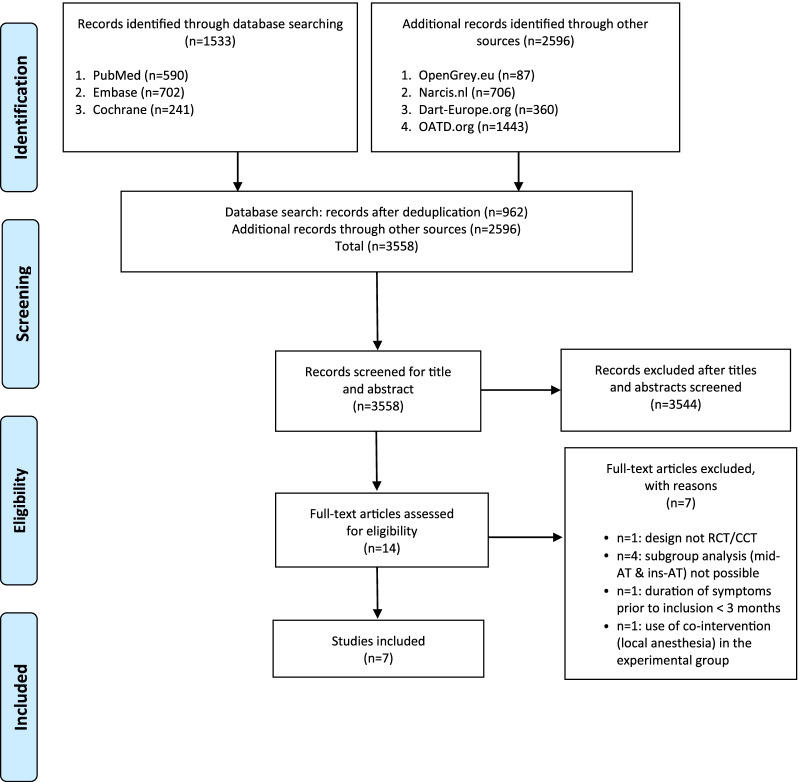
Search strategy

First, the search strategy was applied and all hits were screened on the basis of title and abstract. Eligible studies were then imported into EndNoteX9 and duplicates were removed. Subsequently, full-text studies were obtained and eligibility criteria applied to select studies meeting our research question. The selection process was recorded in a PRISMA flow diagram (Fig. 1).

### Data Collection Process

The following data were extracted from the included studies using a standardized extraction form: (1) authors, (2) year of publication, (3) study design, (4) study population and setting, (5) AT-type (ins-AT and/or mid-AT), (6) duration of symptoms, (7) type of shockwave therapy (F-ESWT or R-ESWT), (8) number of shocks applied, (9) dose of ESWT, (10) number of treatment sessions, (11) treatment duration and frequency, (12) comparisons (e.g., oral medication, injections, surgical or other conservative interventions), (13) outcome measures, (14) length of follow-up, (15) results/conclusions, and (16) industry funding (y/n). For all outcome measures in each study the following data were extracted to facilitate meta-analysis: (a) point estimates of effect: mean differences, risk ratios or odds ratios; (b) estimates of variability: 95% confidence intervals, standard deviations or standard errors; (c) the number of participants; and (d) P-values. In case of missing data, the original authors were contacted for further information.

### Risk of Bias Assessment in Individual Studies

We used the Risk of Bias in Randomized Trials (RoB 2) tool to determine the risk of bias in the primary studies [25]. The RoB 2 assesses risk of bias in 5 distinct domains: (1) bias arising from the randomization process, (2) bias due to deviations from intended interventions, (3) bias due to missing outcome data, (4) bias in measurement of the outcomes, and (5) bias in selection of the reported results. After formulating a risk of bias judgment for each domain, an overall risk of bias judgment was formulated for the outcomes being assessed, and defined as either: ‘low risk,’ ‘some concerns,’ or ‘high risk’ of bias.

### Methodological and Clinical Heterogeneity

A priori we defined subgroups to address methodological and clinical heterogeneity between studies. With regard to the study design, we distinguished RCTs from CCTs, since results of the latter are known to be more susceptible to various kinds of bias [26]. Furthermore, clinical heterogeneity is expected to be introduced by including participants with both AT types in our study. Because mid-AT and ins-AT are considered different clinical entities in the literature [27, 28], we divided them into subgroups.

### Data Syntheses

Collected data were entered in Review Manager (RevMan) 5.4 [29]. If data were clinically and statistically sufficiently homogeneous, we summarized them in a meta-analysis using Random Effects Models (REM) under the assumption that different studies were estimating different, yet related intervention effects (e.g., ESWT-type applied or treatment protocols) [23]. In case fewer than 5 studies were included per AT-type (ins-AT or mid-AT), analyses were performed using Fixed Effect Models (FEM). Continuous outcomes were calculated and expressed as mean difference (MD) or as standardized mean difference (SMD), depending on the similarity of the used scales. Dichotomous data were expressed as relative risk (RR).

In case different scales were used in the reported outcomes (i.e., continuous, categorical, or dichotomous scales), we dichotomized the continuous and categorical scales for our data synthesis. For this, we used the minimal clinically important difference (MCID) as a cutoff point to measure clinically relevant treatment effects. With regard to the VISA-A questionnaire, we considered a decrease of 6.5 points as the MCID [30]. For pain, we incorporated the results of Salaffi et al. [31], in which one point (scale 0–10) or 15% reduction of pain on a NRS represents the MCID for a patient.

We assessed statistical heterogeneity by visually inspecting forest plots for: (1) adequate or poor overlap of 95% confidence intervals (CIs), as poor overlap may be indicative of statistical heterogeneity; and (2) the magnitude and direction of effects. Subsequently, the presence of heterogeneity was statistically determined using the *I*^2^ statistic and classified. We considered a value of less than 40% as an indication of low heterogeneity and a value of 75% or more as an indication of high heterogeneity [23]. In case of heterogeneity, we planned a subgroup analysis and meta-regression analysis to explore possible differences in AT-type, type of ESWT applied, duration of follow-up, or methodological features respectively. Results were presented in a descriptive summary of findings table. We categorized follow-up into short term (≤ 3 months), midterm (3 to 12 months), and long term (≥ 12 months) as previously reported [11].

A priori we planned sensitivity analyses to test the robustness of our results for the impact of removing results from: (1) CCTs, (2) studies with high or unclear risk of bias, and (3) studies that received industry funding.

In case ten or more studies were included in the meta-analysis, we generated a funnel plot for every outcome to assess publication bias [23].

### Grading the Evidence

The Grading of Recommendations Assessment, Development and Evaluation (GRADE) was used to rank the body of evidence [32]. Quality of evidence can be defined as either: ‘high quality,’ ‘moderate quality,’ ‘low quality,’ or ‘very low quality.’ Using the GRADE approach, RCTs start with a ‘high quality’ rating and can be downgraded to ‘moderate quality,’ ‘low quality,’ or ‘very low quality,’ depending on the presence of five factors: (1) risk of bias, (2) inconsistency of results, (3) indirectness of evidence, (4) imprecision, and (5) publication bias. Usually a quality rating will fall down by one level for each factor that is present, up to a maximum of three levels for all factors. In case of major concerns regarding the presence of a factor, the evidence level may fall down by two levels due to that factor alone. Despite the fact that CCTs start with a ‘low quality’ rating, grading upwards to ‘moderate quality’ in case of large treatment effects, or even to ‘high quality’ in case of very large treatment effects, may be warranted if no obvious bias explains these large effects [23].

## Results

### Search Results

Our database search yielded 1533 hits (Fig. 1). After removal of duplicates, the 962 remaining articles were screened for potential inclusion on the basis of title and abstract. We identified 14 studies for full-text review. Among these was one trial protocol [33] that we later included because it was published [34] before submitting this systematic review. Following full-text screening, seven studies were excluded for not meeting our eligibility criteria: In four studies, ESWT was investigated in a mixed cohort from which subgroup analysis for mid-AT and ins-AT separately was not possible [20, 35–37], one study did not meet the required symptom duration prior to inclusion [38], one study was not a (randomized) controlled clinical trial [39], and one study was excluded due to use of local anesthesia in the experimental group [40]. The search resulted in the inclusion of 7 RCTs. Despite the fact that we performed an extensive search for gray literature (Fig. 1), we were not able to retrieve any additional studies. No deduplication was performed for our gray literature search.

### Included Studies

#### Mid-portion Achilles Tendinopathy

We included 3 RCTs meeting our eligibility criteria for mid-AT [21, 41, 42]. Study characteristics, results of primary outcomes, and conclusions are summarized in Table 1.

**Table 1:** Individual study characteristics of the included studies

References	Population and setting, inclusion and exclusion criteria	Experimental group	Control group(s)	Follow-up	Primary outcome, results and conclusions	Industry funding
Rompe et al. [21]	*Population and setting* Primary care setting in Gruenstadt, Germany *Inclusion criteria* 18–70 years mid-AT symptoms ≥ 6 months failure of non-operative management *Exclusion criteria* peritendinous injection within the last 4 weeks bilateral mid-AT symptoms ≤ 6 months concomitant painful ankle conditions congenital or acquired deformities of ankle or knee prior surgery to the ankle or the Achilles tendon prior Achilles tendon rupture prior dislocations or fractures in the area in the preceding 12 months	*R-ESWT (n* = *25)* 2000 pulses, 8 pulses/sec, 3 bar pressure, equals an energy flux density (EFD) of 0.1 mJ/mm^2^, 3 sessions, weekly intervals	*Eccentric loading (n* = *25)* Progressive buildup from 1 set of 10 repetitions to 3 sets of 15 repetitions (1 min rest between sets), twice a day, 7 days a week, for 12 weeks (mild–moderate pain was allowed), starting with body weight and continuing pain-free training with 5 kg rucksack *Wait-and-see (n* = *25)* 1 visit to their orthopedic physician for load management, stretching and ergonomic advice. Pain medication was prescribed if necessary	4 months	VISA-A (range 0–100, mean ± SD) *ESWT* Baseline: 50.3 ± 11.7 4 months: 70.4 ± 16.3 *Eccentric loading* Baseline: 50.6 ± 11.5 4 months: 75.6 ± 18.7 *Wait & see* Baseline: 48.2 ± 9.0 4 months: 55.0 ± 12.9 *Results* No baseline differences between all groups ESWT & eccentric loading improved over time; no differences between treatments The ESWT & eccentric loading groups achieved better VISA-A scores than wait-and-see group *Conclusions* ESWT & eccentric loading showed comparable results at 4 month follow-up. The wait-and-see strategy was ineffective	No potential conflict of interest declared
Rompe et al. [41]	*Population and setting* Primary care setting in Gruenstadt, Germany. Enrollment via orthopedic physician *Inclusion criteria* 18–70 years mid-AT symptoms ≥ 6 months failure of non-operative management *Exclusion criteria* professional athletes peritendinous injection within the last 4 weeks bilateral mid-AT symptoms ≤ 6 months concomitant painful ankle conditions congenital or acquired deformities of ankle or knee prior surgery to the ankle or Achilles tendon prior Achilles tendon rupture prior dislocations or fractures in the area in the preceding 12 months	*Eccentric loading* + *R-ESWT (n* = *34)* Loading consisted of progressive buildup from 1 set of 10 repetitions to 3 sets of 15 repetitions (1 min rest between sets), twice a day, 7 days a week, for 12 weeks (mild–moderate pain was allowed), starting with body weight and continuing pain-free training with 5 kg rucksack R-ESWT consisted of 2000 pulses, 8 pulses/sec, 3 bar pressure (equals EFD 0.1 mJ/mm^2^), 3 sessions for each participant, weekly intervals after 4 weeks of eccentric training	*Eccentric loading (n* = *34)* Progressive buildup from 1 set of 10 repetitions to 3 sets of 15 repetitions (1 min rest between sets), twice a day, 7 days a week, for 12 weeks (mild–moderate pain was allowed), starting with body weight and continuing pain-free training with 5 kg rucksack	4 months	VISA-A (range 0–100, mean ± SD) *Eccentric loading* + *ESWT* Baseline: 50.2 ± 11.1 4 months: 86.5 ± 16.0 *Eccentric loading* Baseline: 50.6 ± 10.3 4 months: 73.0 ± 19.0 *Results* No baseline differences between groups Both groups improved over time Eccentric loading + ESWT achieved better VISA-A scores than eccentric loading alone *Conclusions* At 4 month follow-up, eccentric loading alone was less effective than eccentric loading combined with shockwave treatment	No potential conflict of interest declared
Abdelkader et al. [42]	*Population and setting* Faculty of Physical Therapy in Cairo, Egypt. Referral by the orthopedic department physician *Inclusion criteria* unilateral mid-AT symptoms for ≥ 6 months failure of conservative treatment for at least 3 months *Exclusion criteria* physical therapy or peritendinous injection within the previous 4 weeks use of NSAIDs in the previous week bilateral AT concomitant painful ankle conditions previous injury or surgical treatment to the ankle	*Eccentric loading* + *stretching* + *R-ESWT (n* = *25)* Loading consisted of 3 sets of 15 repetitions (1 min rest between sets), twice a day, seven days a week, for 4 weeks Gastrocnemius and soleus stretches were performed twice a day, 3 repetitions (30 s stretch, 30 s rest) R-ESWT consisted of 2000 pulses, 8 pulses/second, 3 bar pressure (equals EFD 0.1 mJ/mm^2^), 4 sessions, weekly intervals	*Eccentric loading* + *stretching* + *sham R-ESWT* *(n* = *25)* Loading consisted of 3 sets of 15 repetitions (1 min rest between sets), twice a day, seven days a week, for 4 weeks Gastrocnemius and soleus stretches were performed twice a day, 3 repetitions (30 s stretch, 30 s rest) sham-ESWT was administrated in the same way as ESWT. Machine settings were adjusted to generate zero energy, while producing the same sound effect	1 month and 16 months	VISA-A (range 0–100, mean ± SD) *Eccentric loading, stretching & ESWT* Baseline: 24.2 ± 6.5 1 month: 85 ± 6.2 16 months: 80 ± 5.3 *Eccentric loading, stretching & sham-ESWT* Baseline: 21.0 ± 5.2 1 months: 53.4 ± 7.7 16 months: 67 ± 5.6 *Results* Both groups were comparable at baseline Both groups improved over time The experimental group achieved better VISA-A scores than the control group *Conclusion* Adding ESWT to an eccentric loading and stretching program resulted in greater improvements in both the short and long term	No funding
Rompe et al. [45]	*Population and setting* Primary care setting in Gruenstadt, Germany. Enrollment via orthopedic physician *Inclusion criteria* 18–70 years ins-AT ≥ 6 months failure of non-operative management *Exclusion criteria* (imaging) signs of mid-AT, retrocalcaneal bursitis, and Haglund deformity peritendinous injection within the last 4 weeks bilateral mid-AT symptoms ≤ 6 months concomitant painful ankle conditions congenital or acquired deformities of ankle or knee prior surgery to the ankle or Achilles tendon prior Achilles tendon rupture prior dislocations or fractures in the area in the preceding 12 months	*R-ESWT* *(n* = *25)* 2000 pulses, 8 pulses/sec, 2.5 bar pressure (equals EFD 0.12 mJ/mm^2^), 3 sessions, weekly intervals	*Eccentric loading (n* = *25)* Progressive buildup from 1 set of 10 repetitions to 3 sets of 15 repetitions (1 min rest between sets), twice a day, 7 days a week, for 12 weeks (mild to moderate pain was allowed), starting with own body weight and continuing pain-free training with 5 kg rucksack	4 months	VISA-A (range 0–100, mean ± SD) *ESWT* Baseline: 53.2 ± 5.8 4 months: 79.4 ± 10.4 *Eccentric loading* Baseline: 52.7 ± 8.4 4 months: 63.4 ± 12.0 *Results* No baseline differences between groups Both groups improved over time The ESWT group achieved better VISA-A scores than the eccentric loading group *Conclusion* Eccentric loading showed inferior results to ESWT	No funding
Pinitkwamdee et al. [44]	*Population and setting* Orthopedic outdoor clinic in Bangkok, Thailand *Inclusion criteria* 18–70 years clinical or radiographical diagnosis of ins-AT symptoms > 6 months failed other standard conservative care for 3 months (e.g., rest, medication, activity modification, stretching exercise, and heel lift orthosis) *Exclusion criteria* injection to the insertion within the previous 4 weeks mid-AT symptoms neurological deficit history of foot and ankle infection or trauma foot or ankle deformity history of foot or ankle surgery contraindications for ESWT (hemophilia, coagulopathy, or foot and ankle malignancy)	*R-ESWT* + *standard care (n* = *16)* R-ESWT consisted of 2000 pulses, 8–12 Hz, 2.5–3.5 bar pressure (equals EFD 0.12–.16 mJ/mm^2^), 4 sessions, weekly intervals Standard care consisted of rest, medication, activity modification, stretching, and heel lift orthosis	*sham-ESWT* + *standard care (n* = *15)* sham-ESWT was administered by disconnecting the treatment probe while connecting a second probe that generated the shockwave sound (without patient contact) Standard care consisted of rest, medication, activity modification, stretching, and heel lift orthosis	2,3,4,6,12, and 24 weeks	VAS (range 0–10, mean ± SD) *ESWT* + *standard care* Baseline: 6.0 ± 2.6 2 weeks: 4.6 ± 3.1 3 weeks: 3.7 ± 3.0 4 weeks: 2.9 ± 2.2 6 weeks: 3.0 ± 2.3 12 weeks: 2.3 ± 2.5 24 weeks: 2.8 ± 3.3 *sham-ESWT* + *standard care* Baseline: 5.2 ± 2.2 2 weeks: 2.9 ± 1.9 3 weeks: 3.1 ± 2.3 4 weeks: 2.6 ± 2.2 6 weeks: 3.7 ± 2.9 12 weeks: 2.3 ± 2.6 24 weeks: 2.0 ± 2.6 *Results* No baseline differences between groups ESWT showed significant improvements at weeks 4, 6, and 12 sham-ESWT showed significant improvements at weeks 12 and 24 No differences between groups at 24 weeks *Conclusion* There was no difference at 24 weeks with the use of ESWT for chronic insertional Achilles tendinopathy, especially in elderly patients. However, it may provide a short period of therapeutic effects as early as weeks 4 to 12	No funding
Notarnicola et al. [43]	*Population and setting* Hospital in Bari, Italy. Patients were recruited from an orthopedic hospital unit *Inclusion criteria* 18–80 years ins-AT symptoms ≥ 6 months functional VAS score > 4 *Exclusion criteria* (imaging) signs of mid-AT, partial rupture, calcaneal spurs or calcifications contraindications to laser therapy or ESWT (neoplasia, current or previous infections of the affected area, history of epilepsy, coagulopathies, cardiac pacemaker, pregnancy, intolerance to cold) previous Achilles tendon surgery peritendinous injection within the previous 4 weeks ESWT or laser therapy within the previous 2 months congenital or acquired deformities of the lower limb	*F-ESWT* + *eccentric loading* + *stretching (n* = *30)* F-ESWT consisted of 1600 pulses, EFD 0.05–0.07 mJ/mm^2^, 3 sessions, at 3–4 day intervals Eccentric loading consisted of 3 sets of 10 repetitions using a TheraBand (i.e., a thin ribbon of stretchy material that enables resistance during movement exercises), 2–3 weekly sessions for 2 months Calf and Achilles stretching consisted of 4 sets of 15–20 s, 2–3 weekly sessions for 2 months	*Cold air and high-energy laser therapy (CHELT)* + *eccentric loading* + *stretching (n* = *30)* CHELT consisted of simultaneous wavelengths (1,064, 810 and 980 nm; total dosage 1,200 J) together with a flow of cold air (− 30 °C), 10 daily sessions Eccentric loading consisted of 3 sets of 10 repetitions using a TheraBand, 2–3 weekly sessions for 2 months Calf and Achilles stretching consisted of 4 sets of 15–20 s, 2–3 weekly sessions for 2 months	10–15 days (end of complete session of treatment), 2 months, and 6 months	VAS (range 0–10, mean ± SD) *ESWT* + *standard care* Baseline: 7 ± 1.2 10th-15th days: 4.9 ± 0.9 2 months: 5.4 ± 2.7 6 months: 3.3 ± 2.4 *CHELT* + *standard care* Baseline: 7 ± 1.0 10th–15th days: 2.3 ± 1.1 2 months: 2.4 ± 1.6 6 months: 1.7 ± 1.0 *Results* No baseline differences between groups Both groups improved over time CHELT achieved better than ESWT *Conclusion* CHELT gave quicker and better pain relief. It also gave the patient a full functional recovery and greater satisfaction	Not reported
Mansur et al. [34]	*Population and setting* Tertiary teaching hospital in São Paulo, Brazil *Inclusion criteria* 18–75 years pain at the calcaneal tendon insertion for ≥ 3 months diagnosis of ins-AT *Exclusion criteria* bilateral tendinopathy previous surgery autoimmune conditions neuropathy inflammatory diseases non-insertional or mixed tendinopathy previous infiltration pregnancy use of a pacemaker coagulopathies local infection	*R-ESWT* + *Eccentric loading (n* = *58)* R-ESWT consisted of 2000–3000 pulses, 7–10 Hz, and 1.5–2.5 bars of pressure, 3 sessions: at baseline, after two weeks, and after 4 weeks Loading consisted of 3 sets of 15 repetitions with a stretched knee, and 3 sets of 15 repetitions with a 20° flexed knee were performed twice a day, 7 days per week, for 3 consecutive months	*Sham R-ESWT* + *Eccentric loading (n* = *61)* sham-ESWT was administered in the same way as in the experimental group, except that the firing transmission piece was removed from the therapeutic pistol head prior to initiation of ESWT Loading consisted of 3 series of 15 repetitions with a 20° flexed knee, twice a day, 7 days per week, for 3 months	2,4,6,12, and 24 weeks	VISA-A (range 0–100, mean ± SD) *ESWT* + *eccentric loading* Baseline: 43.9 ± 23.2 2 weeks: 43.8 ± 21.3 4 weeks: 50.2 ± 19.6 6 weeks: 49.3 ± 21.2 12 weeks: 53.7 ± 22.0 24 weeks: 63.2 ± 27.5 *sham-ESWT* + *eccentric loading* Baseline: 40.6 ± 21.1 2 weeks: 47.6 ± 19.8 4 weeks: 52.9 ± 20.6 6 weeks: 54.8 ± 19.4 12 weeks: 61.8 ± 23.2 24 weeks: 62.3 ± 25.1 *Results* No baseline differences between groups Both groups improved significantly from baseline No differences between the groups at any time point in the study *Conclusion* ESWT does not potentiate the effects of eccentric strengthening in the management of insertional Achilles tendinopathy	No funding

Rompe et al. [21] randomized participants in three groups, comparing ESWT to eccentric loading, and to a wait-and-see strategy. Eligible secondary outcomes were the NRS for load-induced pain and a Likert scale to evaluate self-perceived recovery. While there were no baseline differences between the groups, patients in the ESWT group and the eccentric loading group achieved significantly better results than patients in the wait-and-see group.

In a second RCT, Rompe et al. [41] compared eccentric loading with additional ESWT to eccentric loading alone. Secondary outcomes were identical to their previous study [21]. There were no baseline differences between the groups. Although both groups improved over time, the ESWT group achieved significantly better results than the eccentric loading group.

In a double-blind RCT by Abdelkader et al. [42], eccentric loading exercises and stretching were performed in the experimental group and the control group. While the experimental group received additional ESWT, sham-ESWT was administrated in the control group. The VAS for pain was the secondary outcome. Although both groups were comparable at baseline and improved over time, the ESWT group achieved better than the sham-ESWT group.

#### Insertional Achilles Tendinopathy

We included 4 RCTs that investigated the effectiveness of ESWT for ins-AT [34, 43–45]. Study characteristics, results of primary outcomes, and conclusions are summarized in Table 1.

Rompe et al. [45] compared ESWT alone to an eccentric loading program [21]. Eligible secondary outcomes were the NRS for load-induced pain and a Likert scale. There were no baseline differences between the groups. While both groups improved, eccentric loading showed inferior results to ESWT.

In a double-blind RCT, Pinitkwamdee et al. [44] compared standard care and ESWT to standard care and sham-ESWT. The secondary outcome was the visual analogue scale foot and ankle (VAS-FA), to evaluate pain and function. The VAS-FA showed no significant difference in outcome between the two groups.

Notarnicola et al.[43] compared standard care with ESWT to standard care and cold air and high-energy laser therapy (CHELT). Secondary outcomes were the ankle–hindfoot scale to evaluate pain and function, and the Roles and Maudsley Score for self-perceived recovery. There were no baseline differences between both groups. While the ankle–hindfoot scale showed significant improvement in both groups, CHELT achieved better than ESWT. Self-perceived recovery only improved significantly in the CHELT group and not in the ESWT group.

Mansur et al. [34] performed a double-blind RCT comparing eccentric exercises and ESWT to eccentric exercises and sham-ESWT. Eligible secondary outcomes were the VAS for pain, the Foot and Ankle Outcome Score (FAOS) to evaluate pain and function, and the 12-item Short Form Health Survey to assess health-related quality of life. Both groups showed significant improvements from baseline in all secondary outcomes with no differences between the groups.

### Risk of Bias Assessment in Included Studies

**Fig. 2:**
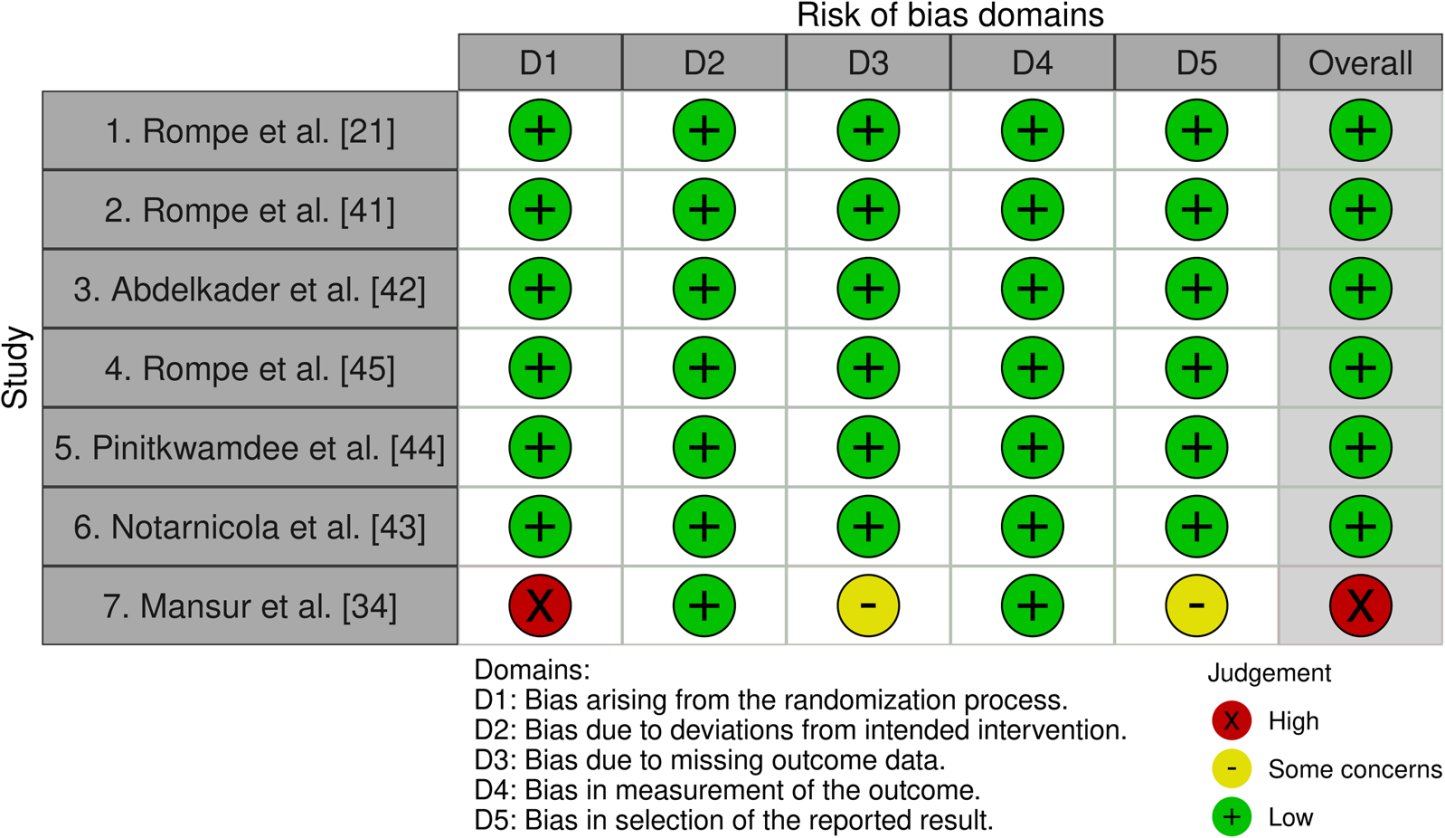
Risk of bias in randomized trials (RoB 2 tool)

Risk of bias was assessed using the RoB2; results are presented in Fig. 2. There were no disagreements between both reviewers.

#### Risk of Bias Arising from the Randomization Process

All three studies on mid-AT [21, 41, 42], and three of the four studies on ins-AT [34, 44, 45] reported using computer-generated numbers in sealed opaque envelopes to draw up an allocation schedule. Allocation was concealed until participants were assigned to an intervention. In the fourth ins-AT study by Notarnicola et al. [43], a stratified randomization procedure was used, aimed at distributing important prognostic variables evenly across both intervention groups. Despite the fact that all studies performed correct randomization procedures, Mansur et al. [34] performed a second randomization procedure due to unforeseen loss to follow-up at week 12. This decision raises concerns as information concerning the procedures followed is lacking, and baseline characteristics are not presented separately for the primary and secondary randomized group. Due to an inappropriate randomization procedure, the risk of bias arising from the randomization process was considered high for this study [34], and low for the other studies [21, 41–45] included.

#### Risk of Bias Due to Deviations from the Intended Interventions

In two studies on mid-AT [21, 41] and two studies on ins-AT [43, 45], blinding participants was not possible due to the obvious nature of the treatments (e.g., eccentric loading, ESWT, or laser therapy). One study on mid-AT [42] and two studies on ins-AT [34, 44] used sham-ESWT in the control groups. It is questionable if performing sham-ESWT always results in complete unawareness of the assigned intervention. For individuals who are familiar with ESWT, the absence of pain or observable shockwaves during treatment may provide some indication of allocation. All studies reported that all randomized participants received the allocated interventions. This has resulted in low risk of bias judgments due to deviations from the intended interventions for all seven studies included.

#### Missing Outcome Data

Two studies on ins-AT [43, 44] and one study on mid-AT [42] reported no loss to follow-up. The remaining two studies on mid-AT [21, 41], and one study on ins-AT [45] reported limited loss to follow-up in the experimental groups, ranging from 4 to 8%. In these studies, baseline values were imputed. Mansur et al. [34] reported a high loss to follow-up, as 13 out of 58 randomized participants (22.4%) in the experimental group discontinued the study. Since the authors did not report the reasons for leaving the study, we cannot exclude the possibility that loss to follow-up was related to participants’ health statuses. A best-case–worst-case scenario was performed for missing data [34]. For this, missing values were imputed for five scenarios, assigning: 0, 25, 50, 75, or 100 points for all missing VISA-A scores. In all cases, the effect was not statistically significant. Due to high loss to follow-up, the risk of bias for missing outcome data was judged to have some concerns for this study [34] and was considered low for the other six studies [21, 41–45] included.

#### Risk of Bias in Measurement of the Outcome

In all three studies on mid-AT [21, 41, 42] and in two studies on ins-AT [34, 45], the VISA-A [46] was used as the primary outcome. The remaining two studies on ins-AT [43, 44] adopted the VAS for pain. Although both instruments are used commonly to evaluate progress in AT [27], the VISA-A questionnaire currently represents the gold standard for the assessment of pain and function [4, 13, 27]. All studies evaluated the experimental and control groups at comparable time points, using the same outcome measures. Six studies [21, 41–45] reported using observer-blinded outcome assessors. Despite the fact that Mansur et al. [34] provided no information on who performed the outcome assessments, blinding was sufficiently executed in their study because a self-completing VISA-A questionnaire was used as primary outcome. Therefore, it is unlikely that this outcome was influenced by knowledge of the intervention received. We considered the risk of bias in measurement of the outcome to be low for all studies [21, 34, 41–45] included.

#### Risk of Bias in Selection of the Reported Result

In all studies, eligible reported results for the outcome domains corresponded to all intended outcome measurements. In six studies [21, 41–45], data were analyzed in accordance with either a trial protocol or a pre-specified statistical analysis plan. Mansur et al. [34] performed a secondary randomization procedure due to unforeseen loss to follow-up at 12 weeks, which they did not state in their trial protocol [33]. This decision may have influenced the outcome as selection bias can occur due to selective loss to follow-up [47]. Moreover, both randomized groups may not be comparable because time period effects may have influenced outcomes [48]. Therefore, the risk of bias in selection of the reported results was judged to have some concerns in this study [34], while in the remaining studies [21, 41–45] this risk was considered to be low.

#### Overall Risk of Bias Judgments in Individual Studies

The overall risk of bias was judged to be low in six studies [21, 41–45] and high in one study [34] (Fig. 2).

### Synthesis of Results

We compared ESWT, either as a monotherapy or as an additional intervention to standard care, to standard care alone. For the purpose of meta-analysis, standard care was defined as conservative care in which at least tendon loading exercises or load management was included. We did not compare ESWT to a wait-and-see strategy, since current literature indicates that all active treatments perform better [5]. Differences in primary outcome measures from baseline to follow-up were defined as treatment effects. For synthesis of results, the study end was used for studies that reported multiple follow-ups [34, 42–44]. With regard to primary outcomes, all studies on mid-AT [21, 41, 42] used the VISA-A questionnaire. Results are therefore presented as MD. Included studies on ins-AT used either the VISA-A [34, 45] or the VAS for pain [43, 44]; hence, results are reported as SMD. For interpretation of the SMD, we applied Cohen’s d [49]: (1) small effect size: SMD 0.2 to  < 0.3, (2) moderate effect size: SMD 0.3 to  < 0.8, and a (3) large effect size: SMD ≥ 0.8. Since less than 10 studies were included in the meta-analysis, we did not generate a funnel plot to assess publication bias.

#### ESWT for Mid-AT

**Fig. 3:**
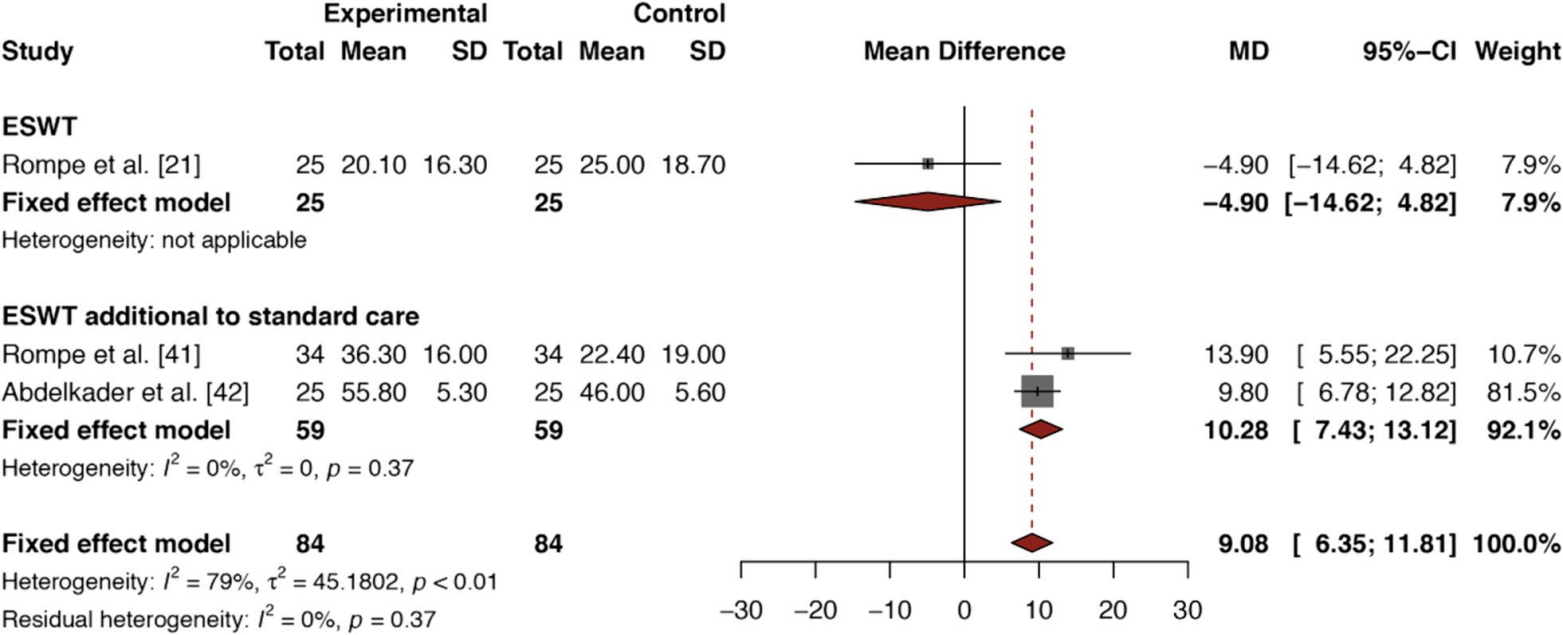
Forest plot of *ESWT versus standard care* for mid-AT, with a subset of *ESWT additional to standard care* versus standard care alone. MD > 0 in favor of experimental intervention

Results are presented in Fig. 3; the intervention characteristics are defined in Table 1. In the first study, Rompe et al. [21] used ESWT as a monotherapy, reporting a small and nonsignificant effect in favor of standard care (MD VISA-A − 4.90, 95% CI − 14.62 to 4.82). The second study of Rompe et al. [41] showed that combining ESWT and standard care was more effective than standard care alone (MD VISA-A 13.90, 95% CI 5.55–22.25). In the third study, Abdelkader et al. [42] concluded that ESWT additional to standard care performed superior to sham-ESWT and standard care (MD VISA-A 9.80, 95% CI 6.78–12.82). Meta-analysis was performed using FEM and resulted in a pooled MD on the VISA-A of 9.08 points (95% CI 6.35–11.81) in favor of ESWT (Fig. 3). An *I*^2^ statistic of 79% was indicative of high (≥ 75%) statistical heterogeneity. Visual inspection of the forest plot (Fig. 3) showed opposite directions of effects and a poor overlap of the 95% CIs, when comparing the first study of Rompe et al. [21] with the second study of Rompe et al. [41] and the study of Abdelkader et al. [42]. In the latter two studies [41, 42], ESWT was used as an additional intervention to standard care, achieving higher VISA-A scores than the first study [21], in which ESWT was administrated as a monotherapy. In order to explore clinically relevant heterogeneity, we created two subsets in R studio [50] (version R-3.6.3), using the packages Meta, Metafor and Readr: (1) ESWT versus standard care and (2) ESWT additional to standard care versus standard care (Fig. 3). Due to apparent differences in outcomes and treatment programs, plural-FEM were used for subgroup analysis. The test for subgroup differences (meta-analytical method: Inverse variance method) indicated a significant (*p* = 0.0033) between-group difference between ESWT versus standard care and ESWT additional to standard care versus standard care. Subgroup analysis of ESWT additional to standard care [41, 42] resulted in a pooled MD on the VISA-A of 10.28 points (95% CI 7.43–13.12). In this subgroup, the *I*^2^ statistic was 0%, whereas the 95% CI showed excellent overlap.

#### ESWT for Ins-AT

**Fig. 4:**
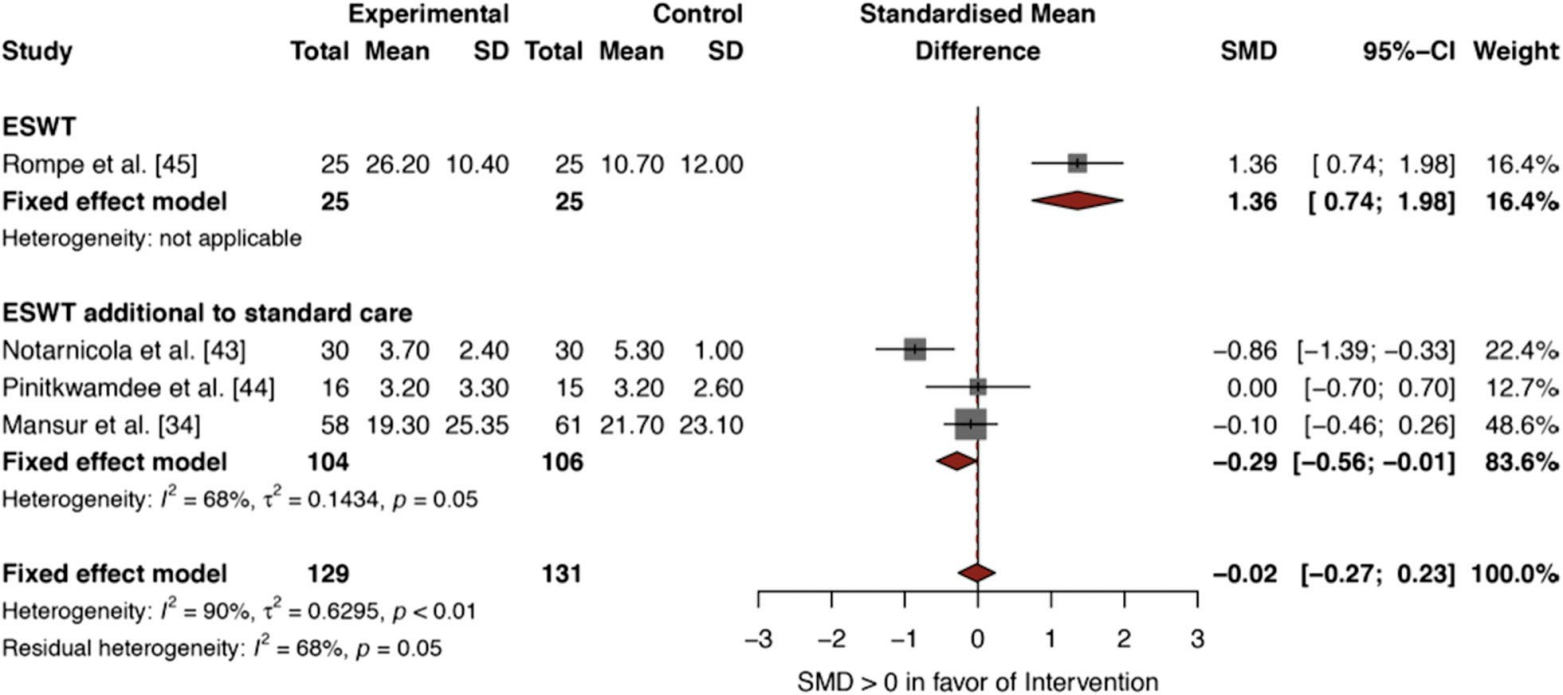
Forest plot of *ESWT versus standard care* for ins-AT, with a subset of *ESWT additional to standard care* versus standard care alone

Results are presented in Fig. 4; the intervention characteristics are defined in Table 1. Rompe et al. [45] reported a positive effect (SMD 1.36, 95% CI 0.74 to 1.98) for ESWT (MD VISA-A 26.20) compared to standard care (MD VISA-A 10.70). This was the only study that evaluated ESWT as a monotherapy. In contrast, Notarnicola et al. [43] reported a significant negative effect (SMD − 0.86, 95% CI − 1.39 to − 0.33) for ESWT additional to standard care (MD VAS 3.70) compared to standard care alone (MD VISA-A 5.30). It should be acknowledged that CHELT was part of the standard care program in their control group.

The remaining two studies [34, 44] presented comparable results. Both Pinitkwamdee et al. [44] and Mansur et al. [34] compared ESWT to sham-ESWT as additional interventions to standard care. Pinitkwamdee et al. [44] found no significant difference (SMD 0.00, 95% CI − 0.70 to 0.70) between the ESWT group (MD VAS 3.20) and the sham-ESWT group (MD VAS 3.20). Mansur et al. [34] also reported no significant difference (SMD − 0.10, 95% CI − 0.46− to 0.26) when comparing ESWT (MD VISA-A 19.30) to sham-ESWT (MD VISA-A 21.70).

Meta-analysis was performed using FEM and resulted in a pooled SMD of − 0.02 (95% CI − 0.27 to 0.23), indicating a not statistically significant negative effect for ESWT (Fig. 4). An *I*^2^ statistic of 90% was indicative of high (≥ 75%) statistical heterogeneity.

Visual inspection of the forest plot showed (Fig. 4) no overlap of the 95% CIs between the study of Rompe et al. [45] and the remaining three studies [34, 43, 44] that used ESWT as an additional intervention to standard care. In order to explore clinically relevant heterogeneity we created two subsets in R studio [50]: (1) ESWT versus standard care, and (2) ESWT additional to standard care versus standard care (Fig. 4). The test for subgroup differences (fixed effect model) indicated a significant (*p* < 0.0001) between-group difference between ESWT versus standard care and ESWT additional to standard care versus standard care (meta-analytical method: Inverse variance method). Quantitative synthesis of the three studies [34, 43, 44] that used ESWT as an additional intervention to standard care resulted in a pooled SMD of − 0.29 (95% CI − 0.56 to − 0.01), indicating a small but statistically significant negative effect of ESWT additional to standard care compared to standard care alone. In this subgroup analysis there was still substantial heterogeneity, as the *I*^2^ statistic was reduced to 68%. In the subgroup, the forest plot showed excellent overlap of the 95% CIs between the studies of Mansur et al. [34] and Pinitkwamdee et al. [44], and to a lesser extent when comparing these studies to the study of Notarnicola et. al. [43].

### Sensitivity Analysis

In our protocol, we planned sensitivity analyses to test the robustness of our results for the impact of removing results from: (1) CCTs; (2) studies with high or unclear risk of bias; and (3) studies that received industry funding. We did not perform a sensitivity analysis for study design since all studies included were randomized controlled trials. Due to a lack of studies, we also did not perform sensitivity analyses for risk of bias and industrial funding, as only one study [34] showed a deviating risk of bias judgment (Fig. 2), and only one study [43] did not declare no conflicts of interest (Table 1).

### Grading the Body of Evidence

GRADE [32] was used to rank the body of evidence for the pooled VISA-A scores of mid-AT and ins-AT. There were no disagreements between both reviewers.

Regarding *risk for bias*, six out of the seven studies included in this systematic review were judged to be at low risk for bias, while in one study on ins-AT [34] the risk was considered high (Fig. 2). Since this study was not likely to seriously alter our results for ins-AT, the evidence levels for both mid-AT and ins-AT were not downgraded for *risk for bias*.

For *inconsistency*, no downgrading was performed for mid-AT since high heterogeneity (*I*^2^ of 79%) [21, 41, 42] was reduced to low heterogeneity (*I*^2^ of 0%) following subgroup analysis of the studies that used ESWT as an additional intervention to standard care [41, 42] (Fig. 3). In contrast, included studies on ins-AT showed varying directions of effect, poor overlap of the 95% CIs, and high heterogeneity (*I*^2^ of 90%) that was still substantial (*I*^2^ of 68%) following subgroup analysis [34, 43, 44] (Fig. 4). Therefore, we downgraded the evidence level for ins-AT to moderate quality of evidence.

Regarding *indirectness*, all studies on mid-AT made direct comparisons of ESWT to standard care, using the VISA-A score to assess pain and function. Moreover eligibility criteria, ESWT interventions and controls for mid-AT were also not indicative of downgrading of the evidence level for mid-AT. Contrastingly, one study on ins-AT [43] did not make a direct comparison between ESWT and standard care, as CHELT was part of the standard care program in the control group. This was the only study to report a statistically significant negative effect for ESWT. We downgraded the evidence level for ins-AT to low quality of evidence on behalf of *indirectness*.

With regard to *imprecision*, we downgraded the evidence for ins-AT to very low quality of evidence as applying the lower and upper boundary of the 95% CI around the pooled estimate would influence the clinical decision-making process. While the lower boundary indicates a negative effect for ESWT (SMD − 0.27), the upper boundary favors ESWT (SMD 0.23) over standard care. Furthermore, we included a relatively small total pooled sample for mid-AT (*n* = 168) and ins-AT (*n* = 260), not meeting the optimal information size of 400 patients (200 per group) for achieving sufficient power in a meta-analysis when pooling continuous data [51]. Therefore, we downgraded the evidence level for mid-AT to moderate quality of evidence, and for ins-AT to very low quality of evidence.

*Publication bias* was not assessed due to a small number of included studies.

In summary, we found moderate quality of evidence to support the effectiveness of ESWT for mid-AT, and very low quality of evidence indicating that ESWT has no additional value over standard care for ins-AT.

## Discussion

To our current knowledge, this is the first meta-analysis that synthesizes evidence from RCTs only to assess the effectiveness of ESWT for mid-AT and ins-AT separately. For mid-AT, we found moderate quality of evidence for the overall effectiveness of ESWT compared to standard care (pooled MD VISA-A 9.08, 95% CI 6.35–11.81) [21, 41, 42]. This effect was mainly attributed to the inclusion of two studies [41, 42] that used ESWT as an additional intervention to standard care, as the remaining study [21] showed a negative, though nonsignificant, effect for ESWT compared to standard care as monotherapies (Fig. 3).

Subgroup analysis to determine the effect of ESWT additional to standard care for mid-AT resulted in a pooled MD on the VISA-A of 10.28 points (95% CI 7.43–13.12) (Fig. 3). These findings are consistent with previous studies [16, 17] and clinical guidelines [4, 13], suggesting that combining ESWT and eccentric exercises may result in superior effectiveness for mid-AT.

For ins-AT, the evidence was more conflicting, as we included two studies [34, 44] that found no significant effect for ESWT over standard care, while the remaining two studies reported a large positive effect [45] and a small negative effect [43] for ESWT, respectively (Fig. 4). Overall, we found very low quality of evidence (SMD − 0.02, 95% CI − 0.27 to 0.23), indicating that ESWT has no added value to standard care for ins-AT (Fig. 4). Subgroup analysis for ESWT additional to standard care for ins-AT even indicated a negative effect (SMD − 0.29, 95% CI − 0.56 to − 0.01) when compared to standard care alone (Fig. 4).

Our results for ins-AT are not supported by two recently published systematic reviews [52, 53] which indicate that adding ESWT to an eccentric loading program increases outcomes for ins-AT. As these two reviews included primary studies with predominantly lower evidence levels such as retrospective and prospective cohort studies, case series, case control studies and pilot studies, this may have contributed to different outcomes compared to our review. Two out of three RCTs [34, 44] in our subgroup analysis on ESWT additional standard care for ins-AT used sham-ESWT in their control groups (Table 1). Double-blinded placebo-controlled studies are more likely to approximate the true effect of ESWT than studies with an observational design. Both trials [34, 44] were double-blind, reporting no significant effect for ESWT over standard care (Fig. 4). In this light, we cannot explain the results of the third trial of the subgroup analysis [45] (Fig. 4), as this was the only study to report a positive effect for ESWT, using a comparable treatment program (Table 1).

Our subgroup analysis on ESWT additional to standard care for ins-AT indicates that adding ESWT to an eccentric loading program results in inferior outcomes (SMD − 0.29, 95% CI − 0.56 to − 0.01) compared to standard care alone (Fig. 4). Caution is warranted when interpreting this pooled estimate, as it is unlikely that ESWT nullifies the effect of a standard care program. Both R-ESWT and F-ESWT have been reported to be safe interventions, with adverse effects such as post-therapy transient skin reddening or discomfort, typically being minor or occurring rarely [18, 19, 54]. Our negative pooled estimate is most likely the consequence of including the study of Notarnicola et al. [43] in our synthesis, being the only study showing a statistically significant negative effect of ESWT for ins-AT (Fig. 4). Notarnicola et al. [43] made no direct comparison between ESWT and standard care (e.g., loading exercises or load management) as high-intensity laser therapy was part of the standard care program in the control group (Table 1). From this study, it is possible to deduce that either high-intensity laser therapy is a superior intervention, or that their ESWT program lacked effectiveness. We cannot substantiate which scenario is most likely applicable. Although laser therapy is widely used to reduce pain and promote tissue healing in multiple healthcare domains, experimental evidence regarding its effectiveness in AT is currently lacking [55, 56]. Randomized controlled studies comparing laser therapy and ESWT have indicated comparable effectiveness in bone healing [57], plantar fasciitis [58], tennis elbow [59], and subacromial pain [60], while reporting a significant advantage for ESWT in treating myofascial pain [61]. Moreover, the ESWT program in the study of Notarnicola et al. [43] differed from all other studies included in this systematic review, as participants received 3 sessions of F-ESWT at 3–4 day intervals, while all other studies included used R-ESWT at either 3 or 4 weekly intervals. To our current knowledge, there is no evidence for superior effectiveness of either R-ESWT or F-ESWT for treating mid-AT or ins-AT. Both modalities are commonly indicated for treating various tendinopathies [11, 19]. Randomized controlled studies have shown that F-ESWT is superior to R-ESWT in treating non-calcific rotator cuff tendinopathies [62] and plantar fasciitis [63], while there appears to be no difference in effectiveness for treating patellar tendinopathy [64] and tennis elbow [65].

Despite the fact that various physiological effects have been attributed to ESWT (e.g., tissue and nerve regeneration, neovascularization, anti-inflammation, anti-apoptosis and a chondroprotective effect), the mechanism of action remains unknown [19]. This makes it particularly difficult to explain why our results indicate that ESWT appears to be effective for treating mid-AT, but not ins-AT, although similar results have been reported for eccentric loading exercises [66]. Mid-AT appears to involve isolated tendon pathology, in contrast to ins-AT [13, 67]. It is possible that ESWT is less effective in treating certain non-tendinous tissues, as ins-AT may be accompanied by metabolic diseases [52], and often includes pathology in adjacent bursae and bone tissue, making the source of pain difficult to diagnose [13, 68, 69]. In particular, intratendinous bone formation in ins-AT is considered difficult to treat [68].

We adopted a MCID of 6.5 points on the VISA-A in order to determine the clinical relevance of outcomes. To date, this score has only been formally established for ins-AT [30]. Most clinical trials investigating the effect of loading exercises in mid-AT use MCIDs ranging up to 20 points, with a change score of 10 points being the most commonly adopted MCID [27]. Included primary studies in this systematic review reported mean improvements in VISA-A scores ranging from 20.1 to 55.8 points for mid-AT (Fig. 3), and 19.30–26.20 points for ins-AT (Fig. 4), while mean improvements for VAS scores for ins-AT ranged from 3.20 to 3.70 points. This should be kept in mind when interpreting our pooled estimates, as we compared ESWT to the standard of care, the latter being defined as a treatment program in which at least tendon loading exercises or load management was included. Since all active treatments for AT are reported to perform better than a wait-and-see policy [5], we chose not to compare ESWT to such a policy, as this would artificially enhance the contrast between treatment arms, most likely resulting in more favorable effects for ESWT.

Regarding primary outcome measures, Pinitkwamdee et al. [44] and Notarnicola et al. [43] used VAS for pain, while all remaining studies included [21, 34, 41, 42, 45] adopted the VISA-A questionnaire (Table 1). Although the latter is considered to represent the gold standard for evaluating the clinical course of AT [4, 13, 27], the VAS and NRS for pain are also commonly used to evaluate progress in these patients [27]. Murphy et al. [27] suggested that pain during a functional task may even be a better measure of immediate treatment effect than the VISA-A questionnaire. The VAS and NRS have been found to be valid, reliable, and responsive in multiple musculoskeletal pain conditions [31, 70–74]. For these reasons, during risk of bias assessment, we did not consider the use of the VAS as primary outcome measure [43, 44] to be inappropriate. Using pain as a primary outcome measure for AT may be debatable, as the VAS and NRS both have not yet been validated in AT [27]. Moreover, apart from associated pain, AT is also known to affect function [1]. Despite the fact that most patients recover from AT, 23 to 37% experience long-term symptoms, lasting up to 10 years [9, 13]. It is possible that in these cases function will improve over time, without significant changes in pain levels. It should be acknowledged that if we had considered the use of the VAS to be inappropriate, this would have resulted in high overall risk of bias judgments for the studies of Pinitkwamdee et al. [44] and Notarnicola et al. [43] (Fig. 2). However, it is unlikely that using the VAS has contributed to inconsistent study outcomes for ins-AT, as Pinitkwamdee et al. [44] and Mansur et al. [34] reported similar results, using the VAS and VISA-A questionnaire as primary outcomes, respectively.

Our pooled estimate for mid-AT was graded *moderate* quality of evidence, while the evidence level for ins-AT was graded *very low* quality of evidence. Because less than 10 studies were included in the meta-analysis, we did not assess publication bias [23]. We decided not to downgrade the evidence level for lack of this assessment, as we performed an extensive search for gray literature, and were unable to find any (ongoing) trials. It is quite possible that only a few controlled studies have been conducted, since ESWT does not represent the state-of-the-art treatment for AT [4, 13].

### Limitations

Our study has several limitations. First, our pooled estimates are most likely not generalizable to individuals unwilling or unable to perform a tendon loading program, as they may represent an underestimation of the true effect of ESWT in contrast to a wait-and-see strategy. This should be taken into account when considering ESWT as a monotherapy for these patients. We found evidence from one high-quality study [21] for mid-AT, and one high-quality study for ins-AT [45], indicating that ESWT is effective as a monotherapy (Table 1). Caution is warranted when generalizing these results, since these were the only studies that evaluated ESWT as a monotherapy. Second, our results may not be adequately generalizable to individuals suffering from combinations of mid-AT and ins-AT, as we aimed to establish the effectiveness of ESWT for mid-AT and ins-AT separately. We excluded studies evaluating the effectiveness of ESWT in mixed cohorts of mid-AT and ins-AT if it was not possible to perform a subgroup analysis. Although both tendinopathies are considered to be different clinical entities in the literature, they can coexist [27, 28].

## Conclusion

The findings of this systematic review indicate that adding ESWT to a tendon loading program in mid-AT results in a clinically important improvement on the VISA-A. Our findings cannot support the use of ESWT for ins-AT, with two double-blind RCTs [34, 44] indicating that this treatment is ineffective. Although we were able to include several recently published studies, the availability of controlled studies, eligible to answer our review question, appears limited at present. It should be emphasized that the number of RCTs included in this systematic review was limited, and the pooled sample of mid-AT and ins-AT patients was relatively small. Future high-quality RCTs are needed to support our findings.

## Supplementary Information

**Additional file 1**. Database searching.

## Data Availability

All data generated or analyzed during this study are included in this published article and its supplementary information files.

## Abbreviations

AT: Achilles tendinopathy
ESWT: Extracorporeal shockwave therapy
Mid-AT: Mid-portion Achilles tendinopathy
Ins-AT: Insertional Achilles tendinopathy
RCTs: Randomized controlled trials
CCTs: Controlled clinical trials
F-ESWT: Focused extracorporeal shockwave therapy
R-ESWT: Radial extracorporeal shockwave therapy
MD: Mean difference
SMD: Standardized mean difference
PRISMA: Preferred reporting items for systematic reviews and meta-analyses guidelines
PROSPERO: International prospective register of systematic reviews
VISA-A: Victorian Institute of Sports Assessment—Achilles questionnaire
NRS: Numeric rating scale for pain
VAS: Visual analogue scale for pain
UMC: University Medical Center
RoB 2: Risk of Bias in randomized trials
REM: Random effects models
FEM: Fixed effect models
MCID: Minimal clinically important difference
RR: Relative risk
CIs: Confidence intervals
GRADE: Grading of recommendations assessment, development and evaluation
VAS-FA: Visual analogue scale foot and ankle
CHELT: Cold air and high-energy laser therapy
FAOS: Foot and ankle outcome score
EFD: Energy flux density
SD: Standard deviation
