# Supplementary material for: Extracorporeal Shockwave Therapy for Mid-portion and Insertional Achilles Tendinopathy: A Systematic Review of Randomized Controlled Trials
Source: Sports Med Open. 2022 May 13;8:68. doi: 10.1186/s40798-022-00456-5 (PMC9106789; doi:10.1186/s40798-022-00456-5)
Supplement: Supplementary file 1 — Additional file 1. Database searching. [file 40798_2022_456_MOESM1_ESM.docx]

Appendix I

Searches: Faridi van Etten, Amsterdam UMC, Medical Library AMC.

21-1-2021:

| Databases: |  |  |
| --- | --- | --- |
| PubMed/Medline, Embase (Ovid), Cochrane Central | Before deduplication | After deduplication |
| Total | 1533 | 962 |

PubMed

590 hits:

("Achilles Tendon"[Mesh] OR "Tendinopathy"[Mesh] OR "Tendon Injuries"[Mesh] OR "Tendons"[Mesh] OR achilles[tiab] OR tendoachilles[tiab] OR calcaneal[tiab] OR tendinopath*[tiab] OR tendon patholog*[tiab] OR tendon injur*[tiab] OR tendinos*[tiab] OR tendinitis[tiab]) AND ("Extracorporeal Shockwave Therapy"[Mesh] OR shockwave*[tiab] OR shock wave*[tiab] OR extra corporeal pulse-activ*[tiab] OR EPAT[tiab])

EMBASE (OVID)

Database(s): Issue: Embase Classic+Embase 1947 to 2021 January 20
Search Strategy:

| **#** | **Searches** | **Results** |
| --- | --- | --- |
| 1 | achilles tendon/ | 11120 |
| 2 | exp tendinitis/ | 18932 |
| 3 | exp tendon injury/ | 24755 |
| 4 | exp tendon/ | 49312 |
| 5 | (achilles or tendoachilles or calcaneal or tendinopath* or tendinos* or tendinitis).ti,ab,kw. | 30806 |
| 6 | (tendon* adj3 (patholog* or injur*)).ti,ab,kw. | 6900 |
| 7 | 1 or 2 or 3 or 4 or 5 or 6 | 94903 |
| 8 | shock wave therapy/ | 1531 |
| 9 | (shockwave* or shock wave* or extracorporeal pulse activ* or EPAT).ti,ab,kw. | 15979 |
| 10 | 8 or 9 | 16242 |
| 11 | 7 and 10 | 702 |

Abbreviations: exp, “explodes” controlled vocabulary term (e.g., expands search to all more specific related terms in the vocabulary’s hierarchy).

[Cochrane Central Register of Controlled Trials](https://www.cochranelibrary.com/)

Issue 1 of 12, January 2021

ID Search Hits

#1 (achilles or tendoachilles or calcaneal or tendinopath* or tendinos* or tendinitis):ti,ab,kw 2730

#2 (tendon* near/3 (patholog* or injur*)):ti,ab,kw 659

#3 #1 or #2 3085

#4 (shockwave* or shock wave* or extracorporeal pulse activ* or EPAT):ti,ab,kw 2593

#5 #3 and #4 in Trials 241
